# Single-Chamber Microbial Fuel Cell with an Innovative Sensing Component for Real-Time Continual Monitoring of a Wide Range of Cr(VI) Concentrations in Wastewater

**DOI:** 10.3390/bios15030158

**Published:** 2025-03-03

**Authors:** Guey-Horng Wang, Jong-Tar Kuo, Chiu-Yu Cheng, Ying-Chien Chung

**Affiliations:** 1 Research Center of Natural Cosmeceuticals Engineering, Xiamen Medical College, Xiamen 361008, China; 2 Department of Biological Science and Technology, China University of Science and Technology, Taipei 115, Taiwan

**Keywords:** chromate reductase gene, cyclic voltammetry, hexavalent chromium, microbial fuel cell, polarization curve

## Abstract

Hexavalent chromium (Cr(VI)) is toxic, carcinogenic, and harmful to biological systems. Common detection methods, such as colorimetry, atomic absorption spectrometry, ion chromatography, and biological systems, can only be used in the laboratory and do not provide real-time feedback. To address these limitations, the current study cloned the *ChrB* gene, which exhibits high specificity in detecting Cr(VI), and the *ChrA* gene, which exhibits high Cr(VI) tolerance, into *Escherichia coli*. This recombinant strain, *ChrA–ChrB–E. coli*, was integrated into a single-chamber microbial fuel cell for accurate continual monitoring over a wide range of Cr(VI) concentrations. *ChrA–ChrB–E. coli* thrived in temperatures from 25 °C to 45 °C and pH levels between 5 and 8. Its ability to reduce Cr(VI) remained consistent across Cr(VI) forms, carbon sources, and oxyanions. Cyclic voltammetry was employed to verify the electrical activity of the biosensor. The biosensor exhibited a detection limit of 0.0075 mg/L. Under conditions simulating the regulatory emission limit for Cr(VI) of 0.5 mg/L in industrial wastewater, the biosensor achieved a response time of 20 s during continual operation. When tested with synthetic wastewater containing Cr(VI) concentrations from 0.02 to 150 mg/L, the system exhibited high adaptability and facilitated stable monitoring (relative standard deviation ≤ 2.7%). Additionally, the biosensor’s accuracy (−1.73% to 2.5%) matched that of traditional batch methods, highlighting its suitability for real-time Cr(VI) monitoring in aquatic environments.

## 1. Introduction

The toxicity, persistence, and bioaccumulation of heavy metals pose serious risks to soil, surface water, food, animals, and human health [1,2]. Hexavalent chromium (Cr(VI)) is a highly toxic heavy metal ion that causes carcinogenic, teratogenic, and mutagenic effects [3]. Specifically, Cr(VI) causes respiratory diseases, lung cancer, and kidney and liver damage and has been placed on the Hazards Priority List of the U.S. Agency for Toxic Substances and Disease Registry [4,5]. Nevertheless, its extensive use in stainless steel production, metal finishing, textile dyeing, leather tanning, and wood preservation [6] renders a complete ban of Cr(VI) impractical. Consequently, methods must be developed to manage the wastewater containing Cr(VI) generated by these processes [7]. In addition to reducing Cr(VI) use and treating wastewater, devices to continually monitor Cr(VI) that are accurate and real-time must be developed to ensure safety [8].

Current methods for detecting Cr(VI) comprise spectrophotometry, atomic absorption spectrometry (AAS), ion chromatography, inductively coupled plasma, electrochemical methods, fluorescence methods, and biological approaches [9,10,11]. These methods have several limitations, such as high equipment costs, substantial consumable requirements, complex pretreatment processes, and a requirement for highly trained operators [12,13,14]. Additionally, the practical applications of these approaches are restricted by their inability to specifically measure Cr(VI) rather than total Cr concentrations and their limited sensitivity and selectivity, narrow detection ranges, and lack of real-time monitoring capabilities [12,13,14].

Transdisciplinary research in biosensor development has led to the development of a promising approach to continual online Cr(VI) monitoring: microbial fuel cells (MFCs) [15]. MFCs typically consist of an anode and a cathode, which may be housed in the same or separate chambers. The configuration of MFC-based biosensors is contingent upon the specific infrastructure and microbial species employed [16]. MFCs have the potential to function as both power supply devices and biosensors [4].

Xu et al. (2015) developed a flat microliter, membrane-based MFC sensor designed for batch operations, which successfully detected Cr(VI) concentrations ranging from 5 to 20 mg/L. Their findings indicated a negative linear association between the levels of Cr(VI) and the voltage output generated by the sensor [17]. In a subsequent study, Chung et al. (2016) created a dual-chamber MFC that utilized Cr(VI) as an electron acceptor in the cathode chamber, enabling the detection of Cr(VI) concentrations between 0.1 and 15 mg/L in batch mode [18]. Furthermore, Wang et al. (2016) inoculated the anode chamber of a dual-chamber MFC with *Ochrobactrum anthropi*, which led to the identification of two distinct linear relationships between voltage output and Cr(VI) concentrations, specifically within the ranges of 0.0125 to 0.3 mg/L and 0.3 to 5 mg/L [16]. Lastly, Wu et al. (2017) inoculated the anode chamber of a dual-chamber MFC with *Exiguobacterium aestuarii*, achieving the precise detection of Cr(VI) concentrations from 2.5 to 60 mg/L [19].

Wu et al. (2019) developed a three-stage, single-chamber MFC (SCMFC) that broadened the detection range of Cr(VI) to between 5 and 90 mg/L, facilitating continuous operation [8]. Furthermore, Lazzarini Behrmann et al. (2020) utilized *Pseudomonas veronii* 2E as an inoculum in an SCMFC to enable the real-time monitoring of Cr(VI) concentrations ranging from 4 to 18.5 mg/L [20]. Moreover, Chang et al. (2023) developed a membraneless laminar flow microfluidic MFC that detected Cr(VI) concentrations from 0.1 to 10 mg/L during continual operation [21]. SCMFCs have low internal resistance, high cathodic oxygen reduction rates, high proton diffusion coefficients, and reduced setup and operating costs [22]. They also require no catholyte or aeration and are simple to use. Hence, they have considerable potential for use in continual online Cr(VI) monitoring.

MFC-based biosensors with optimal detection sensitivity, a wide detection range, and a low detection limit for Cr(VI) require the use of specific microbial species [23]. Strains such as *Arthrobacter aurescens*, *Bacillus cereus*, *Cupriavidus metallidurans*, *Exiguobacterium aurantiacum*, *Lactococcus lactis*, *Leucobacter salsicius*, *Microbacterium* sp., *Micrococcus* sp., *Ochrobactrum anthropi*, and *Serratia* sp. have demonstrated the ability to detect Cr(VI) [24,25,26,27]. Two classes of genes are associated with Cr(VI) removal or detection [28]. The *ChrA* gene, a transporter gene, moves intracellular Cr(VI) through the sulfate channel to the cell exterior [29]. Strains possessing the *ChrA* gene, such as *C. metallidurans*, *E. aestuarii*, *Microbacterium* sp., and *Serratia* sp., can survive in environments with high Cr(VI) concentrations [24]. By contrast, the *ChrB* gene reduces Cr(VI) to other chromium oxidation states [30]. Strains with the *ChrB* gene, such as *O. anthropi* and *O. tritici*, are well-suited for use as biosensor components [24,31]. Several studies have utilized recombinant *Escherichia coli* expressing *ChrA* or *ChrB* genes for luminescent assays to detect trace Cr(VI) levels (0.0005–0.75 mg/L or 0.002–0.75 mg/L) in water in batch operations [32,33].

In this study, we engineered two recombinant genes in *E. coli*: *ChrB* (for high Cr(VI) specificity) and *ChrA* (for high Cr(VI) tolerance). Unprecedentedly, the *E. coli* strain harboring these two recombinant genes was inoculated into an SCMFC as the biorecognition element. A strong T7 promoter was incorporated to enhance this biorecognition element. Additionally, the SCMFC’s low internal resistance and high oxygen rate improved the detection range, lowered the detection limit, and enabled accurate real-time monitoring.

## 2. Materials and Methods

### 2.1. Materials

*Ochrobactrum anthropi* YC152 and *Exiguobacterium aestuarii* YC211 were obtained from the laboratory of Professor Chung [16,19]. *Escherichia coli* BL21(DE3) was acquired from Novagen, located in Darmstadt, Germany. All culture media, biochemical reagents, and chemicals were sourced from Sigma-Aldrich in St. Louis, MO, USA, unless otherwise indicated.

### 2.2. Gene Cloning, Genetic Transformation, and Biosensor Construction

*O. anthropi* and *E. aestuarii* were cultured in lysogeny broth (LB) supplemented with Na_2_Cr_2_O_7_ (50 mg/L as Cr(VI)). The cultures were agitated at 180 rpm and maintained at 30 °C. After 48 h, the culture medium was centrifuged at 6000 rpm to separate the cell pellet. Chromosomal DNA from *O. anthropi* and *E. aestuarii* was extracted and purified using an EP021 Bacteria DNA Extraction Kit (ELK Biotechnology Co., Ltd., Denver, CO, USA). The primers *Nco I-ChrA-f*, *Xho I-ChrA-r*, *Nco I-ChrB-f*, and *Not I-ChrB-r* were used to amplify the *ChrA* and *ChrB* gene fragments through polymerase chain reaction (PCR). The amplified fragments were digested with the restriction enzymes *Nco I*, *Xho I*, and *Not I* (Table 1) [34].

**Figure 1:**
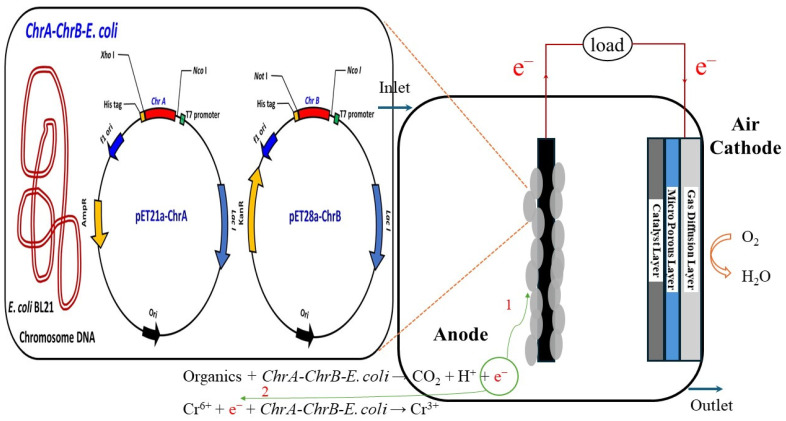
Single-chamber microbial fuel cell (SCMFC)-based biosensor along with its biosensing component, specifically highlighting the *ChrA–ChB–E. coli* sensor in an enlarged view on the left. ^1^ Wastewater containing organic matter is introduced into the SCMFC, where the strain *ChrA–ChB–E. coli* oxidizes it to produce e^−^. These electrons travel through the external circuit to the cathode, generating electricity. ^2^ If Cr^6+^ is also present in the wastewater, *ChrA–ChB–E. coli* will utilize the e- derived from the oxidation of organic matter to reduce Cr^6+^ to Cr^3+^. This process results in a diminished flow of electrons to the cathode, consequently leading to a decrease in electricity generation. The linear relationship between the Cr^6+^ concentration in the effluent and the corresponding voltage of SCMFC allows this system to function effectively as a Cr^6+^ biosensor.

The total volume of the polymerase chain reaction (PCR) was established at 50 μL, comprising 35 μL of deionized water (dH_2_O), 5 μL of 10× PCR buffer, 4 μL of deoxynucleotide triphosphates (dNTPs), 2 μL of each primer set (at a concentration of 10 ng/μL), 1 μL of template DNA (at a concentration of 10 pmol), and 1 μL of Taq DNA polymerase. The PCR protocol commenced with an initial denaturation step at 95 °C for 10 min, followed by 30 cycles of amplification. Each cycle included a denaturation phase at 95 °C for 30 s, an annealing phase at 55 °C for 30 s, and an extension phase at 72 °C for 90 s. A final elongation step at 72 °C for 7 min was performed to ensure the completion of DNA synthesis. The PCR products corresponding to the *ChrA* and *ChrB* genes were subsequently ligated into the pET21a and pET28a vectors, which had been previously digested with the same restriction enzymes, utilizing T4 DNA ligase at 16 °C. The resultant recombinant plasmids were designated as pET21a-ChrA and pET28a-ChrB. Competent *E. coli* BL21(DE3) cells were transformed with either pET21a-ChrA or pET28a-ChrB or both constructs. For the transformation process, 10 pg of plasmid DNA was combined with competent *E. coli* cells in a microcentrifuge tube, gently mixed for 3 s, and incubated on ice for 5 min. The mixture was then subjected to heat shock at 42 °C for 45 s. Following this, 400 μL of LB medium was added, and the cells were incubated at 37 °C for 1 h [35]. A 100 μL aliquot of the diluted transformed cells was subsequently spread onto an LA medium supplemented with 150 μg/mL ampicillin, 12.5 μg/mL kanamycin, or a combination of both antibiotics and incubated at 35 °C for 20 h. Antibiotic concentrations ensured the presence of equal amounts of pET21a-ChrA and pET28a-ChrB in the *E. coli*. The resulting recombinant strains were designated as *ChrA–E. coli* (control group), *ChrB–E. coli* (control group), and *ChrA–ChrB–E. coli* (Figure 1).

To verify the success of the transformation, we cultured *ChrA–ChB–E. coli* in LB medium supplemented with 150 µg/mL of ampicillin and 12.5 µg/mL of kanamycin. After 18 h, plasmid extraction was conducted using an EasyPure Plasmid DNA Mini Kit (Bioman Scientific Co., Ltd., New Taipei City, Taiwan). Subsequently, electrophoresis was conducted to confirm the presence of the plasmid under the following parameters: a 1.2% agarose gel subjected to 200 V for 12 min, utilizing 0.5× Tris-acetate EDTA buffer.

### 2.3. Evaluation of the Growth Rate of ChrA–ChB–E. coli 

In this study, the *E. coli* (control group) and *ChrA–ChrB–E. coli* strains were inoculated at a concentration of 5 × 10^6^ cfu/mL into Erlenmeyer flasks containing 200 mL of LB medium. The pH of the culture was maintained at 7.0, the incubation temperature was set at 35 °C, and the stirring rate was set to 180 rpm. Quantitative samples of the culture were collected at regular intervals to measure the optical density at 600 nm (OD_600_) until the growth curve reached a plateau. Additionally, the specific growth rate (μ) during the logarithmic growth phase of both bacterial strains was calculated.

### 2.4. Adaptability of ChrA–ChrB–E. coli to Environmental Conditions

#### 2.4.1. Tolerance of *ChrA–ChrB–E. coli* to Varying Cr(VI) Concentrations

The original strain of *ChrA–ChrB–E. coli* exhibited the ability to eliminate Cr(VI). This experiment assessed whether this ability was retained following the transfer of genes. In the experiment, three control strains—*E. coli*, *E. aestuarii*, and *O. anthropic*—were cultured alongside the recombinant strain *ChrA–ChrB–E. coli* in LB medium to achieve a bacterial concentration of 5 × 10^6^ cfu/mL. Subsequently, the cultures were inoculated into LB medium containing varying concentrations of Cr(VI) ranging from 0 to 300 mg/L, with a culture pH of 7.0, a temperature of 35 °C (for *E. coli* and *ChrA–ChrB–E. coli*) or 30 °C (for *E. aestuarii* and *O. anthropic*), and a stirring rate of 180 rpm. After an incubation period of 24 h, the OD_600_ of each culture medium was recorded. The inhibition coefficient was calculated as the ratio of the OD_600_ of the culture medium containing Cr(VI) to that of the culture medium devoid of Cr(VI).

#### 2.4.2. Effects of Temperature and pH on Cr(VI) Reduction by *ChrA–ChrB–E. coli*

The strains *ChrA–ChrB–E. coli* and *E. coli* were cultured in an LB medium at a temperature of 35 °C, with agitation at 180 rpm for a duration of 9 h. Following this incubation period, the culture medium was centrifuged at 6000 rpm for 10 min, after which the supernatant was discarded. The bacterial cells were then resuspended in Tris-buffered mineral salts medium (TMM), achieving a concentration of 5 × 10^6^ cfu/mL. Subsequently, a 10 mL aliquot of this bacterial suspension was inoculated into 200 mL of LB medium that was supplemented with 50 mg/L of Cr(VI) for further cultivation. The effect of temperature on the removal of Cr(VI) by *ChrA–ChrB–E. coli* was investigated within a temperature range of between 15 °C and 45 °C, with a culture duration of 24 h. Furthermore, the effect of pH on the removal of Cr(VI) by *ChrA–ChrB–E. coli* was evaluated by preparing the LB medium with a buffer solution, which was adjusted to a pH range from 4 to 9 with a culture duration of 24 h.

#### 2.4.3. Effect of Carbon Source on Cr(VI) Removal by *ChrA–ChrB–E. coli*

*ChrA–ChrB–E. coli*, at a concentration of 5 × 10^6^ cfu/mL, was introduced into 200 mL of TMM medium that was augmented with Cr(VI) concentrations ranging from 0.5 to 100 mg/L, alongside glucose concentrations varying from 30 to 300 mg/L. After a 24 h incubation period, the efficiency of Cr(VI) removal by *ChrA–ChrB–E. coli* was assessed to clarify the relationship between the concentrations of Cr(VI), glucose, and the corresponding removal efficiency. In the event that the impact was not substantial, it was prudent to investigate the influence of alternative carbon sources, including fructose, formic acid, acetic acid, citric acid, and acetone, on the Cr(VI) removal efficiency by *ChrA–ChrB–E. coli*. Such an assessment can provide a foundational reference for the development of analytical calibration curves in subsequent research.

#### 2.4.4. Effects of Forms of Cr(VI), Cations, and Anions on Cr(VI) Removal by *ChrA–ChrB–E. coli*

*ChrA–ChrB–E. coli* (5 × 10^6^ cfu/mL) was cultured in 200 mL of TMM medium, which was supplemented with varying concentrations of Cr(VI) ranging from 0.05 to 100 mg/L, along with 30 mg/L of glucose. These specific concentrations of Cr(VI) were chosen to replicate the quality of wastewater containing Cr(VI) at different levels. The sources of Cr(VI) employed in this investigation included sodium dichromate (Na_2_Cr_2_O_7_) and sodium chromate (Na_2_CrO_4_). Following a 24 h incubation period, the ability of *ChrA–ChrB–E. coli* to reduce Cr(VI) was assessed. The experimental parameters were maintained at a pH of 7, a temperature of 35 °C, and a stirring rate of 180 rpm. To further investigate the influence of various anions and cations on the ability of *ChrA–ChrB–E*. *coli* to reduce Cr(VI), additional experiments were performed in 200 mL of TMM medium containing Cr(VI) concentrations (0.05–100 mg/L), 30 mg/L of glucose, and different anions and cations at concentrations of 0.5 or 5 mg/L (cations: Cu^2+^, Mn^2+^, Fe^2+^, Cd^2+^, Cr^3+^; anions: SO_4_^2−^, PO_4_^3−^, AsO_4_^3−^). After a 24 h cultivation period, the ability of *ChrA–ChrB–E. coli* to reduce Cr(VI) was evaluated to determine whether the presence of common wastewater contaminants affected the activity, specificity, or selectivity of *ChrA–ChrB–E. coli* in the reduction of Cr(VI). If a notable influence was identified, other pretreatment technologies or reduction strategies were explored in future experiments.

### 2.5. Construction of the Single-Chamber, Microbial Fuel Cell-Based Biosensor

The SCMFC-based biosensor was fabricated using acrylic material with a cubic configuration with dimensions of 5 × 5 × 5 cm^3^, resulting in a working volume of 64 mL (Figure 1). The anode was fabricated from graphite felt and had a surface area of 18 cm^2^. The air cathode consisted of three distinct layers and was manufactured as described in an earlier study [36]. The innermost layer served as the catalytic layer and was composed of carbon cloth integrated with Pt (0.5 mg/cm^2^) and 30% polytetrafluoroethylene (PTFE). The intermediate layer was microporous, had a thickness of 50 μm, and consisted of the conductive material Vulcan XC-72, PTFE, surfactant, and isopropyl alcohol as a preservative. The outermost layer facilitated gas diffusion and was constructed from Sigracet S35 and 5% PTFE. The cathode and anode were connected using silver-plated copper wire, which contained a variable resistor within the circuitry. A pipeline was established above the SCMFC to facilitate the inflow of wastewater, the introduction of inoculants, and the measurement of water quality. Additionally, a pipeline was constructed below the SCMFC to enable outflow and further water quality assessment. The inlet wastewater containing Cr(VI) was initially deoxygenated through the introduction of nitrogen gas. Subsequently, the wastewater was introduced into the SCMFC at a constant flow rate facilitated by a peristaltic pump as air diffused naturally to the cathode. Various electrochemical characteristics were continually monitored using a multimeter (Model 2700, Keithley Instruments, Inc., Solon, OH, USA) connected to an external computer.

To immobilize the *ChrA–ChrB–E. coli* cells within the SCMFC, we introduced an LB culture solution containing 50 mg/L of Cr(VI) and 5 × 10⁶ cfu/mL of *ChrA–ChrB–E. coli*. An external resistance of 1500 Ω was applied. The culture medium was delivered to the SCMFC by using a peristaltic pump with a liquid retention time (LRT) of 10 days, during which the solution was internally circulated to promote the natural adhesion of the *ChrA–ChrB–E. coli* cells to the graphite felt of the anode. When the voltage output of the SCMFC decreased to 10% of its maximum value, the original solution was replaced with a fresh sterile LB culture medium containing 50 mg/L of Cr(VI). This replacement occurred without circulation at an LRT of 2 h. Stabilization of the voltage output confirmed successful immobilization of the *ChrA–ChrB–E. coli* biofilm. Each experiment was conducted in triplicate to ensure the reliability of the average results.

### 2.6. Operating Characteristics of the SCMFC-Based Biosensor

#### 2.6.1. Optimal Operating Resistance

To determine the optimal operating resistance of the biosensor, a TMM medium supplemented with 50 mg/L of Cr(VI) and 30 mg/L of acetic acid was introduced into the SCMFC-based biosensor in a batch operation. Throughout the operation, the external resistance was systematically increased from 50 to 10,000 Ω. Each resistance condition was sustained until the voltage output stabilized for a minimum of 30 min, after which the current and voltage values were recorded to elucidate the characteristics of the SCMFC-based biosensor. This method enabled the identification of the optimal external resistance, which served as a critical parameter for subsequent continual operation experiments.

#### 2.6.2. Optimal Liquid Flow Rate or LRT

Under the conditions of optimal external resistance, we continuously introduced a TMM medium supplemented with 30 mg/L of acetic acid and varying concentrations of Cr(VI) (0.5–200 mg/L) into the SCMFC-based biosensor, progressing sequentially from lower to higher concentrations. The operational interval between each inlet concentration was maintained at a minimum of 2 h. Additionally, we administered the aforementioned solutions with varying LRTs (0.5–4 min). During the experimental procedure, the liquid flow was adjusted from a slow to a rapid rate, with a minimum interval of 30 min between each operational group. After we introduced the solution, we recorded the data and averaged the results over an introduction period varying from 8 to 10 min. The primary objective of this experiment was to identify the optimal LRT that produced a stable voltage output from the SCMFC-based biosensor.

#### 2.6.3. Optimal Stable Time or Response Time

Under the conditions of optimal external resistance, we gradually introduced a TMM medium supplemented with 30 mg/L of acetic acid and varying concentrations of Cr(VI) (0.05–200 mg/L) into the SCMFC-based biosensor with a 2 min LRT. The voltage output of the SCMFC was recorded to assess the duration required to achieve a stable voltage output from the biosensor.

#### 2.6.4. Electrochemical Testing

Electrochemical investigations were performed using cyclic voltammetry (CV) by following the procedure of an earlier study [37]. Initially, a sterile TMM medium supplemented with 30 mg/L of acetic acid and 50 mg/L of Cr(VI), a TMM medium supplemented with *ChrA–ChrB–E. coli* and acetic acid, and a TMM medium supplemented with *ChrA–ChrB–E. coli*, acetic acid, and Cr(VI) were continuously introduced into the SCMFC-based biosensor over the course of 1 week. The experimental conditions were maintained at an external resistance of 800 Ω and an LRT of 2 min. Subsequently, 3 mL aliquots from each solution were subjected to analysis utilizing a CHI 627C multichannel potentiostat from CH Instrument in Austin, TX, USA. The scan rate was established at 30 mV/s, and the analysis was performed across a potential range of +1.0 to −1.0 V. The electrochemical configuration employed a three-electrode system, wherein the anode and cathode acted as the working and counter electrodes, respectively, while the Ag/AgCl electrode (saturated KCl, 222 mV vs. SHE) served as the reference electrode.

### 2.7. Relationship Between Cr(VI) Concentration and Voltage Output of the SCMFC-Based Biosensor in Continual Operation

The SCMFC must exhibit a wide detection range to ensure it can monitor whether wastewater complies with the legally mandated maximum concentration levels of Cr(VI). Hence, this experiment used an external resistance of 800 Ω and an LRT of 2 min as baseline conditions. TMM medium supplemented with varying concentrations of Cr(VI) (0.005–200 mg/L) and an acetic acid concentration of 30 mg/L was gradually administered to the SCMFC. Following exposure to the medium for 4 min, the output voltage of the SCMFC-based biosensor was recorded to establish a correlation between the concentration of Cr(VI) and the output voltage.

### 2.8. Measurement of Cr(VI) in Synthetic Wastewater Under Continual Operation of the Biosensor

To assess the reproducibility and accuracy of the SCMFC-based biosensor in detecting Cr(VI) in varying concentrations in influent wastewater, we prepared a TMM culture medium supplemented with 30 mg/L of acetic acid and varying concentrations of Cr(VI) to create synthetic wastewater. This wastewater was continuously fed into the SCMFC at an LRT of 2 min. We conducted two experiments: The first involved maintaining a constant concentration of Cr(VI) for 3 consecutive days, followed by 1 day of exposure to a Cr(VI)-free culture medium, with this cycle repeated until the 41st day. The second experiment involved varying the concentrations of Cr(VI) on a daily basis to evaluate the adaptability of the SCMFC to varying Cr(VI) concentrations (e.g., during days 20–25 and days 45–55). In addition to employing the SCMFC-based biosensor for continual Cr(VI) measurement, standard detection methods, such as 1,5-diphenylcarbazida (DPC) colorimetry [38], AAS (U.S. Environmental Protection Agency, 1983) [39], and ion chromatography [40], were utilized in batch operations and for off-site analyses, respectively. To validate the analytical methods employed, several validation parameters were evaluated. The linearity of these methods was determined by constructing a calibration curve for Cr(VI) and examining the residuals produced from the linear regression analysis of the response to various concentrations, utilizing the least-squares approach. The detection limit was determined by evaluating the instrument’s response to ten repetitions of blank samples from each matrix under investigation. This comprehensive approach elucidated the differences in Cr(VI) detection between the SCMFC-based biosensor and established methodologies. The voltage readings obtained from the SCMFC were calibrated to convert them into concentrations of Cr(VI).

### 2.9. Statistical Analysis

All experiments were conducted in triplicate at least, and the results are reported as means with standard deviations. The data were analyzed using a one-way analysis of variance (ANOVA), followed by Duncan’s multiple range test for post hoc analysis. A statistical significance threshold was established at *p* < 0.05. All statistical analyses were performed utilizing SPSS version 26 (SPSS, Chicago, IL, USA).

## 3. Results and Discussion

### 3.1. Construction of Recombinant E. coli

To confirm DNA recombination, the plasmid of the recombinant *ChrA–ChB–E. coli* was extracted. Because the purity of an extract may heavily influence subsequent DNA analysis, the absorbance of the extract in the present study was determined at wavelengths of 230, 260, and 280 nm. Typically, the ratio of absorbances at these wavelengths is used as a measure of nucleic acid purity [41]. The OD_260/280_ and OD_260/230_ ratios of the extract were 1.92 and 2.082, respectively. Therefore, this sample met the established quality criteria [39].

**Figure 2:**
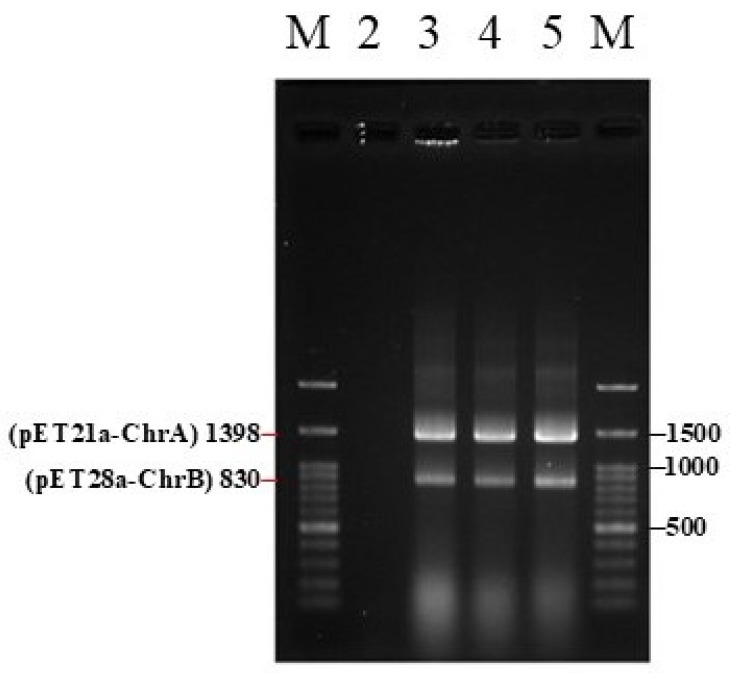
DNA electropherogram of the pET21a-ChrA and pET28a-ChrB recombinant genes (lines 1 and 6 are DNA markers (M), lines 3–5 are triplicate results).

Figure 2 illustrates a DNA electropherogram of the pET21a-ChrA and pET28a-ChrB recombinant genes. The size of the pET21a-ChrA gene was 1398 base pairs (bp) containing a T7 promoter, a T7 terminator, the *ChrA* gene, and a partial sequence of the pET21a plasmid. By contrast, the size of the pET28a-ChrB gene was 830 bp, and it consisted of a T7 promoter, a T7 terminator, the *ChrB* gene, and a partial sequence of the pET28a plasmid. In Figure 2, the presence of bright bands derived from the PCR product near 1500 bp and 800 bp confirmed the construction of the sensing component (*ChrA–ChB–E. coli*).

### 3.2. Characteristics of the Recombinant ChrA–ChB–E. coli Strain

**Figure 3:**
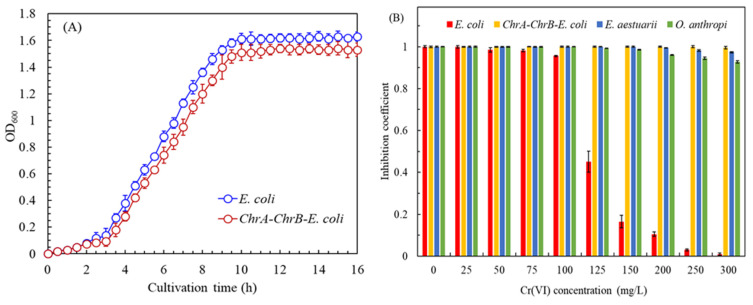

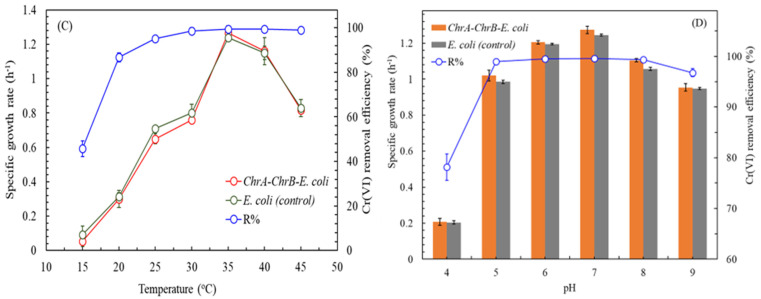
(**A**) Growth curve of *ChrA–ChB–E. coli*. (**B**) Tolerance of *ChrA–ChB–E. coli* to varying Cr(VI) concentrations. (**C**) Adaptability of *ChrA–ChB–E. coli* to changes in temperature. (**D**) Adaptability of *ChrA–ChB–E. coli* to pH.

Figure 3A depicts the growth curve of *ChrA–ChB–E. coli*. The experimental results indicate that both *E. coli* and the recombinant strain *ChrA–ChrB–E. coli* exhibited a log growth phase from hour 3 to hour 10. The specific growth rates (μ) of *E. coli* and *ChrA–ChrB–E. coli* were 1.304 ± 0.015 h^−1^ and 1.285 ± 0.035 h^−1^, respectively. The results of the statistical analysis revealed no significant differences between the two groups (*p* > 0.05). The results also indicated that the growth rate of *E. coli* remained unaffected following DNA recombination. The specific growth rate of *ChrA–ChrB–E. coli* was significantly higher than that of the Cr(VI)-resistant bacterium E. aestuarii with the ChrA gene (μ: 0.470 h^−1^) [19] and the Cr(VI)-reducing bacterium *O. anthropic* with the ChrB gene (μ: 0.482 h^−1^) [16]. This result indicates that the *ChrA–ChrB–E. coli* developed in this study is suitable for industrial applications. On the basis of the growth curve of *ChrA–ChrB–E. coli*, we selected the late logarithmic growth phase (beginning at the 9th hour after cultivation) as the appropriate inoculation time for subsequent experiments.

Figure 3B illustrates the tolerance of *ChrA–ChrB–E. coli* to varying Cr(VI) concentrations. The control group comprised *E. coli*, *E. aestuarii* (the source of the *ChrA* gene), and *O. anthropic* (the source of the *ChrB* gene). The results indicate that a concentration of 125 mg/L of Cr(VI) inhibited the growth rate of *E. coli* by 54.9%. Furthermore, a Cr(VI) concentration exceeding 250 mg/L nearly completely inhibited the growth of *E. coli*. By contrast, the recombinant strain *ChrA–ChrB–E. coli* exhibited a high tolerance to elevated concentrations of Cr(VI). Notably, even at a concentration of 300 mg/L, the effect of Cr(VI) on the growth rate of *ChrA–ChrB–E. coli* was minimal—only 0.5%. *E. aestuarii* exhibited the second-highest tolerance to Cr(VI), with a 2.6% reduction in the growth rate observed at 300 mg/L of Cr(VI). In summary, the recombinant *ChrA–ChrB–E. coli* exhibited a greater tolerance to Cr(VI) concentrations than the original strains (*E. aestuarii* and *O. anthropic*) derived from the transgenic bacteria did.

Figure 3C illustrates the adaptability of *ChrA–ChB–E. coli* to changes in temperature. The results demonstrate that the adaptability of *E. coli* and *ChrA–ChrB–E. coli* to temperature changes was comparable. The optimal growth temperature for both strains was 35 °C. At this temperature, the specific growth rates were 1.240 ± 0.04 h^−1^ for *E. coli* and 1.267 ± 0.02 h^−1^ for *ChrA–ChrB–E. coli*. Furthermore, the effect of temperature on the recombinant and nonrecombinant strains was nonsignificant (*p* > 0.05). Additionally, the temperature range from 25 °C to 45 °C influenced the specific growth rate of *ChrA–ChrB–E. coli*, with specific growth rates from μ 0.65 ± 0.01 to 1.267 ± 0.02 h^−1^. However, the effect of this growth on the efficiency of *ChrA–ChrB–E. coli* in reducing Cr(VI) was weak, with efficiency values ranging from 95.2% ± 1.2% to 99.5% ± 0.25%. These findings underscore the utility of the recombinant *ChrA–ChrB–E. coli* strain as a biosensing component to detect Cr(VI) with a high level of environmental adaptability. This adaptability may be due to the robust expression of the Cr(VI) gene within *ChrA–ChrB–E. coli* [32].

Figure 3D depicts the adaptability of *ChrA–ChB–E. coli* to pH. The findings indicate that the effect of pH on both the recombinant and nonrecombinant bacteria was comparable. The variations in the specific growth rates of both *E. coli* and *ChrA–ChrB–E. coli* at a pH value between 5 and 8 exhibited only slight changes. The specific growth rate of *E. coli* was measured from 0.985 ± 0.01 to 1.245 ± 0.005 h^−1^, whereas the specific growth rate of *ChrA–ChrB–E. coli* ranged from 1.02 ± 0.03 to 1.274 ± 0.02 h^−1^. Under a pH between 5 and 8, the effect of pH on the efficiency of *ChrA–ChrB–E. coli* in reducing Cr(VI) to other Cr oxidative states was not statistically significant, with a reduction efficiency of 99% ± 0.05% to 99.6% ± 0.04%. This result demonstrates the strong adaptability of *ChrA–ChrB–E. coli* to environmental changes when utilized as a biosensing component to detect Cr(VI). This finding indicates that *ChrA–ChrB–E. coli* can be used to monitor Cr(VI) across diverse aquatic environments.

### 3.3. Effects of Coexisting Compounds on the Removal Efficiency of Cr(VI) by the Recombinant ChrA–ChB–E. coli Strain

Various organic substances frequently coexist in aquatic environments with the target compound of Cr(VI). The diversity and concentration of these organic compounds may influence the sensitivity of the components of a biosensor, such as *ChrA–ChB–E. coli*, in detecting Cr(VI) [42]. This study employed glucose as a model carbon source [43] with two concentrations of glucose: a low concentration of 30 mg/L and a high concentration of 300 mg/L. The findings indicate that the glucose negligibly influenced the efficiency of *ChrA–ChrB–E. coli* in removing Cr(VI), which remained consistently high (between 99.8% and 100%) regardless of the concentration of Cr(VI). We further investigated the effects of alternative carbon sources, such as fructose, formic acid, acetic acid, citric acid, and acetone, on the Cr(VI) removal efficiency by *ChrA–ChrB–E. coli*.

**Figure 4:**
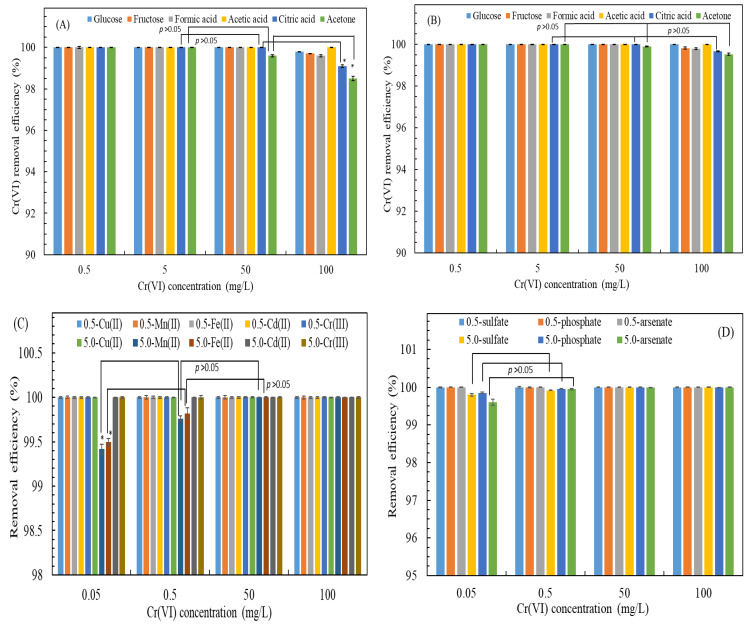
Effects of (**A**) types of carbon sources at 30 mg/L; (**B**) types of carbon sources at 300 mg/L; (**C**) coexisting cations at 0.5 or 5.0 mg/L; and (**D**) coexisting anions at 0.5 or 5.0 mg/L on the Cr(VI) removal efficiency by recombinant *ChrA–ChrB–E. coli*. Data are expressed as the means and standard deviations of three independent experiments. A significant difference is expressed by * *p* < 0.05.

Figure 4A depicts the effects of varying concentrations of the various carbon sources on the Cr(VI) removal efficiency by the recombinant *ChrA–ChrB–E. coli*. The results indicate that at a concentration of 30 mg/L, acetone and citric acid weakly influenced the removal of 100 mg/L Cr(VI). Specifically, the efficiency of *ChrA–ChrB–E. coli* in removing Cr(VI) was reduced slightly to a range from 98.5% ± 0.1% to 99.1% ± 0.06% under both carbon sources (*p* values < 0.05). This reduction in efficiency may reflect metabolic challenges posed by these carbon sources to *ChrA–ChrB–E. coli* [44]. By contrast, at a carbon source concentration of 300 mg/L, the type of carbon source used had a minimal effect on the Cr(VI) removal efficiency (*p* values > 0.05) (Figure 4B). Even when acetone—which resists decomposition—was used, *ChrA–ChrB–E. coli* achieved a removal efficiency of 99.53% ± 0.05%. This result suggests that higher carbon source concentrations enhance Cr(VI) reduction. Because carbon sources function as electron donors in the biochemical reactions of *ChrA–ChrB–E. coli*, carbon sources that facilitate these reactions or are easily degradable support Cr(VI) reduction to other Cr oxidative states [27,45].

This study also assessed the impact of various forms of Cr(VI) (specifically, chromate, CrO_4_^2−^, and dichromate, Cr_2_O_7_^2−^) on the Cr(VI) removal efficiency by *ChrA–ChrB–E. coli*. The results indicate that within a Cr(VI) concentration range of 0.05 to 50 mg/L, the specific form of Cr(VI) utilized as the source had a minimal effect on the removal efficiency. However, at a concentration of 100 mg/L of Cr(VI), a slight reduction in the removal efficiency was noted (from 99.92% ± 0.025% to 99.95% ± 0.028%). Figure 4C presents the influence of coexisting cations and their respective concentrations on the Cr(VI) removal efficiency of *ChrA–ChrB–E. coli* across various Cr(VI) concentrations. The findings demonstrate that the presence of 5 mg/L of Fe(II) or Mn(II) in conjunction with 0.05 mg/L of Cr(VI) significantly inhibited Cr(VI) removal (*p* values < 0.05). Conversely, the introduction of 2 × 10^−5^ M EDTA as a pretreatment to alleviate this interference resulted in an enhancement in the Cr(VI) removal efficiency, increasing from 99.42% ± 0.03% to 99.82% ± 0.06%. Figure 4D illustrates the effects of the coexisting anions and their concentrations on the Cr(VI) removal efficiency of *ChrA–ChrB–E. coli*. The results indicate that the presence of 5 mg/L of sulfate (SO_4_^2−^), phosphate (PO_4_^3−^), or arsenate (AsO_4_^3−^) alongside 0.05 mg/L of Cr(VI) had a negligible impact on the removal efficiency (*p* values > 0.05). In conclusion, the findings suggest that the presence of coexisting substances in wastewater has a minimal effect on the ability of *ChrA–ChrB–E. coli* to reduce Cr(VI).

### 3.4. Determination of the Optimal Operational Parameters for the SCMFC-Based Biosensor

When *ChrA–ChB–E. coli* was introduced as a sensing component into the SCMFC, a progressive and incremental increase in the voltage output of the system was observed. Specifically, the voltage increased from 689 ± 57 mV on the 4th day of operation to 1523 ± 52 mV by the 5th day. Subsequently, the voltage rose gradually, achieving a peak voltage of 1608 ± 51 mV on the 6th day, which was maintained until the 8th day. Subsequently, a gradual decline in voltage occurred, reaching 153 ± 25 mV by the 13th day. This voltage level, which represents one-tenth of the maximum output, suggested a deficiency in the nutrient solution. In consideration of this, we introduced a fresh sterile culture solution. We subsequently observed a gradual increase in the SCMFC voltage, which stabilized at 1089 ± 31 mV between the 16th and 20th days. This stabilization indicated that the system had immobilized the sensing components in the anode of the SCMFC [46].

**Figure 5:**
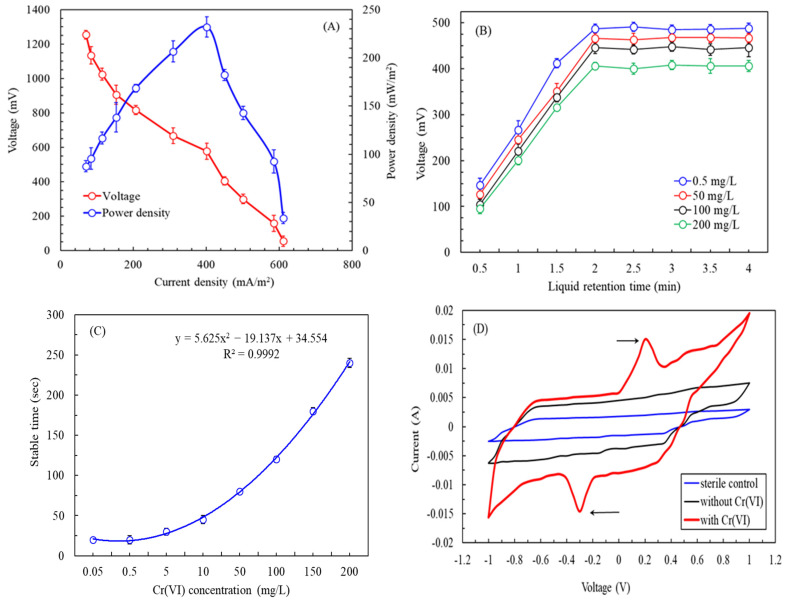
(**A**) Polarization and power density curves obtained from the SCMFC-based biosensor inoculated with *ChrA–ChB–E. coli* (anolyte: TMM medium supplemented with 50 mg/L of Cr(VI) and 30 mg/L of acetic acid; external resistance: 50–10,000 Ω). (**B**) Effects of the liquid retention time on the voltage output of SCMFC-based biosensor in continual operation under varying concentrations of Cr(VI) (anolyte: TMM medium supplemented with 30 mg/L of acetic acid and varying concentrations of Cr(VI); external resistance: 800 Ω). (**C**) Effects of the Cr(VI) concentration on the stable/response time of the SCMFC-based biosensor at 2 min LRT during continual operation (anolyte: TMM medium supplemented with 30 mg/L of acetic acid and varying concentrations of Cr(VI); external resistance: 800 Ω). (**D**) Cyclic voltammetry curves of the SCMFC-based biosensor (LRT: 2 min; external resistance: 800 Ω).

After immobilizing the sensing components, we evaluated the optimal resistance of the SCMFC. Figure 5A illustrates the polarization and power density curves obtained from the SCMFC-based biosensor. The results indicate that the voltage of the SCMFC decreased as the current density increased. Additionally, the power density initially increased with the current density before decreasing. The turning point occurred at a current density of 401.4 ± 4.2 mA/m^2^ (corresponding to an external resistance of 800 Ω). At this point, the voltage measured 578 ± 46.8 mV, and the power density was 232 ± 10.5 mW/m^2^. The external resistance was equal to the internal resistance, indicating that the system’s optimal operating resistance was 800 Ω [47]. This result compares favorably with those in the literature. For example, Wang et al. (2016) conducted an experiment utilizing *O. anthropi* in a dual-chamber MFC and identified an optimal operating resistance of 270 Ω, yielding a voltage of 281 ± 10.6 mV and a power density of 89.1 ± 3.7 mW/m^2^ [16]. In a separate study, Wu et al. (2019) employed *E. aestuarii* in a single-chamber MFC and reported an optimal operating resistance of 500 Ω, a voltage of 367.2 ± 42.5 mV, and a power density of 167.5 ± 6.8 mW/m^2^ [8]. Ali et al. (2024) employed a dual-chamber microbial fuel cell with N, S co-doped carbon nanofibers in a self-standing cathode to reduce Cr(VI); a maximum power density of 155 ± 0.3 mW/m^2^ was observed [48]. These differences in the performance of the MFCs are attributable to differences in MFC configuration, the bacterial species utilized, and the composition of the anolyte in each study. Notably, our system demonstrated the highest power density among the evaluated MFCs. Our findings indicate that the MFC developed in this study strongly amplifies electrical signals, enhancing its potential for use in biosensors.

Figure 5B depicts the effects of the LRT on the voltage output of the SCMFC-based biosensor in continual operation under varying Cr(VI) concentrations. Regardless of the Cr(VI) concentration used, increasing the LRT or reducing the liquid flow rate improved the voltage output. At an LRT of 2 min or longer, the voltage output stabilized, demonstrating that 2 min was sufficient to detect multiple Cr(VI) concentrations. By contrast, shorter LRTs provided insufficient contact time for *ChrA–ChrB–E. coli* to metabolize acetic acid, limiting the electron release necessary for voltage generation [49,50]. Additionally, higher inlet Cr(VI) concentrations resulted in reductions in the SCMFC voltage. For example, the introduction of 0.5 mg/L Cr(VI) resulted in a voltage of 487 ± 10.2 mV, whereas 50 mg/L, 100 mg/L, and 200 mg/L yielded voltages of 466.5 ± 9.8 mV, 446 ± 12.5 mV, and 405.8 ± 8.1 mV, respectively. This reduction occurred because higher Cr(VI) concentrations at the anode enhanced electron theft during the reduction reaction, restricting electron transfer to the cathode through the external circuit [16]. Figure 1 depicts the associated reaction mechanism and equation.

Figure 5C illustrates the effects of the Cr(VI) concentration on the stable/response time of the SCMFC-based biosensor at a 2 min LRT during continual operation. The voltage output was previously measured over 8–10 min at a 2 min LRT. To assess the biosensor’s real-time monitoring, the present experiment was undertaken. Increasing Cr(VI) concentrations lengthened the stable/response time required for accurate measurement. A stable/response time of 20 s was sufficient at low concentrations (0.05–0.5 mg/L). At concentrations between 5 and 100 mg/L, the maximum stable/response time was 2 min. At concentrations of between 150 and 200 mg/L, an LRT of up to 4 min was required. Accurate measurements within an LRT of 4 min were facilitated by introducing a wastewater concentration of between 0.05 and 200 mg/L of Cr(VI) into the SCMFC. For wastewater containing 0.5 mg/L Cr(VI), the maximum discharge concentration for industrial wastewater, measurements were achieved in 20 s, meeting the requirements for real-time monitoring. This efficiency stems from the strong ability of *ChrA–ChrB–E. coli* to remove Cr(VI) [51]. The approach proposed in the current study is considerably superior to the result obtained in the literature, in which SCMFCs with *E. aestuarii* required a minimum detection time of 200 s for low Cr(VI) concentrations at a 2 min LRT [8]. These findings underscore the advantages of using *ChrA–ChrB–E. coli* as a sensing component to detect Cr(VI).

CV is used to examine microbial activity in MFCs by analyzing redox peak intensity, which reflects biofilm electrochemical activity [52]. Figure 5D depicts the CV curves of the SCMFC-based biosensor. Notably, no redox couple was observed for the sterile culture medium or the medium without Cr(VI) but containing *ChrA–ChrB–E. coli*. However, a redox couple was observed for the medium containing both Cr(VI) and *ChrA–ChrB–E. coli*, with an oxidation peak at 201 mV and 15 mA and a reduction peak at −306 mV and −14.6 mA during the reverse scan. The pronounced voltametric signals indicated strong bio-electrochemical activity [53]. Because *E. coli* is typically electrically inactive [54], this activity likely resulted from the Cr(VI) resistance gene (*ChrA*) or the Cr(VI) reductase gene (*ChrB*) in the recombinant *ChrA–ChrB–E. coli* strain [34]. Another study also suggested that the *ChrA* membrane protein may facilitate conduction [55].

### 3.5. Relationship Between Cr(VI) Concentration and the Voltage Output of the SCMFC-Based Biosensor in Continual Operation

**Figure 6:**
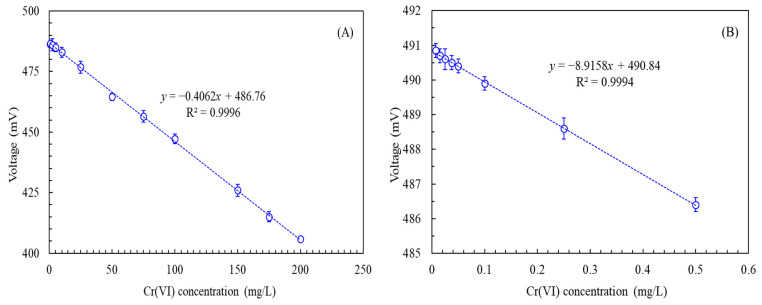
Relationship between the Cr(VI) concentration and voltage output of the SCMFC-based biosensor in continual operation: (**A**) Cr(VI) concentration: 0.5 to 200 mg/L; (**B**) Cr(VI) concentration: 0.0075 to 0.5 mg/L (operational temperature: 35 °C, external resistance: 800 Ω, anolyte: TMM supplemented with 30 ppm of acetic acid and varying concentrations of Cr(VI), response time: 4 min).

A regression was conducted to examine the relationship between concentrations of Cr(VI) from 0.005 to 200 mg/L and the output voltage of the SCMFC-based biosensor. The results revealed a linear relationship characterized by a coefficient of determination R^2^ of 0.957. Nevertheless, the relationship between the concentration and the output voltage exhibited a larger significant deviation at lower Cr(VI) concentrations. To refine our analysis, we employed stepwise regression, which yielded two optimal linear ranges for the concentration and output voltage: 0.5–200 mg/L, represented by the regression equation *y* = −0.4062*x* + 486.76, and 0.0075–0.5 mg/L, represented by the regression equation *y* = −8.9158*x* + 490.84 (Figure 6). The R^2^ for both linear relationships exceeded 0.999, indicating a high degree of correlation, and the detection limit was 0.0075 mg/L. The SCMFC system was capable of continually monitoring the Cr(VI) concentration in water in real time through the use of these equations.

Past research employed *O. anthropi* in a double-chamber MFC to detect Cr(VI) concentrations of between 0.0125 and 5 mg/L in batch operations [16]. *E. aestuarii* was also used to detect Cr(VI) concentrations of between 2.5 and 60 mg/L in a double-chamber MFC under batch conditions [19] and concentrations of between 5 and 90 mg/L in a three-stage SCMFC system under continual operation [8]. The T7-lux *E. coli* biosensor, which uses T7 as the promoter, was employed to analyze diluted Cr(VI) concentrations of between 0.0005 and 0.5 mg/L in batch operations [32]. A fluorescent method employing gold nanoclusters was also used to measure Cr(VI) concentrations from 0.01 to 10 mg/L [56], and a colorimetric Cu(II)-GMP system was employed to determine concentrations from 0.052 to 1.3 mg/L [57]. These results highlight the advantages of the SCMFC-based biosensor developed in the present study, specifically its broad detection range, low detection limit, and facilitation of continual real-time monitoring.

### 3.6. Cr(VI) Measurement in Synthetic Wastewater Using the SCMFC-Based Biosensor in Continual Operation

**Figure 7:**
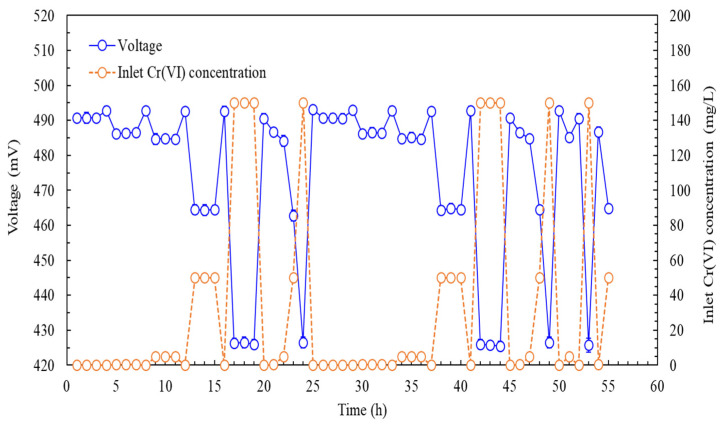
Continual Cr(VI) measurement in synthetic wastewater using the SCMFC-based biosensor inoculated with *ChrA–ChrB–E. coli* at an LRT of 2 min (external resistance: 800 Ω, anolyte: TMM supplemented with 30 mg/L of acetic acid and varying concentrations of Cr(VI)).

To evaluate the SCMFC-based biosensor’s performance in continually monitoring Cr(VI) concentrations under varying disturbance conditions, we varied inlet Cr(VI) concentrations to test the biosensor’s feasibility in wastewater applications. Figure 7 depicts voltage fluctuations from the SCMFC-based biosensor inoculated with *ChrA–ChrB–E. coli* at different inlet Cr(VI) concentrations in synthetic wastewater. The voltage output decreased as the Cr(VI) concentration increased. At 0 mg/L of Cr(VI), the biosensor achieved a maximum stable output voltage of 492.8 ± 0.5 mV on days 4, 8, 12, and 16. Between days 20 and 24, a rapid increase in the Cr(VI) concentration from 0.02 mg/L to 150 mg/L resulted in a large decrease in voltage from 490.65 ± 0.51 mV to 425.86 ± 1.06 mV. From days 26 to 44, a gradual increase in the Cr(VI) concentration from 0.02 mg/L to 150 mg/L resulted in a consistent voltage decline. Additional experiments assessed short-term Cr(VI) increases (days 45–49) and fluctuations (days 50–55). Despite these variations, the biosensor maintained stable performance. At consistent inlet Cr(VI) concentrations of 0.02 mg/L, 0.5 mg/L, 5 mg/L, 50 mg/L, and 150 mg/L, the biosensor’s average voltages were 490.65 ± 0.51 mV, 486.41 ± 0.32 mV, 484.74 ± 0.51 mV, 465.96 ± 0.82 mV, and 426.89 ± 1.12 mV, respectively. The relative standard deviation (RSD) values ranged from 0.64% to 2.7%, indicating minor potential for biofouling or performance drift. By contrast, the luminescent *E. coli* biosensor for Cr(VI) batch monitoring exhibited higher RSD values of between 3.2% and 3.6% [32]. These results indicate the SCMFC-based biosensor’s superior stability, reproducibility, and performance, indicating its suitability for continual Cr(VI) detection in synthetic wastewater.

To compare the SCMFC-based biosensor with standard methods for Cr(VI) detection in synthetic wastewater, we used AAS, colorimetric methods, and ion chromatography in batch operations because these conventional approaches do not enable continual monitoring. The average voltage recorded by the SCMFC-based biosensor (Figure 7) was converted into Cr(VI) concentrations by using the standard curve illustrated in Figure 6. Table 2 presents the Cr(VI) concentrations measured using these methods. In batch analysis, ion chromatography resulted in the smallest deviations, ranging from −0.36% to 3.8%. By contrast, the results of an AAS analysis exhibited a high RSD of 6.5% at 0.02 mg/L due to the proximity of this concentration to the instrument’s detection limit [39]. The colorimetric method, with detection ranges of 0.01–0.1 mg/L and 0.1–1 mg/L, required the use of dilution to measure Cr(VI) concentrations between 5 and 150 mg/L, which may lead to increased errors [38]. Ion chromatography, with a detection limit of 0.3 µg/L, performed well at low Cr(VI) concentrations [40]. Nevertheless, under continual monitoring, the SCMFC-based biosensor yielded RSDs of between −1.73% and 2.5%, with minimal variation across a wide range of Cr(VI) concentrations. These results surpassed those of the standard methods, indicating that the biosensor developed in the present study enables accurate and continual monitoring of electroplated wastewater containing Cr(VI), which typically has few additional pollutants. Further studies are required to evaluate this SCMFC’s performance with other wastewater types containing Cr(VI) that involve complex matrices and diverse pollutants.

## 4. Conclusions

This study developed an electrochemical device with a novel *ChrA–ChrB–E. coli*-sensing component to enable rapid Cr(VI) detection in aqueous environments. *ChrA–ChrB–E. coli* offers higher specificity and tolerance to Cr(VI) than other bacterial strains that reduce Cr(VI) to other oxidative states of Cr. *ChrA–ChrB–E. coli* also exhibits a faster growth rate, higher specificity, and enhanced tolerance to Cr(VI). *ChrA–ChrB–E. coli* adapts well to diverse environmental conditions, reducing Cr(VI) across broad pH and temperature ranges and in the presence of various carbon sources, metal cations, and oxyanions. The SCMFC system features low internal resistance and a high cathodic oxidation rate that provides high sensitivity and an acceptable Cr(VI) detection range of concentrations from 0.0075 to 200 mg/L. Under conditions simulating the regulatory emission limit for Cr(VI) of 0.5 mg/L in industrial wastewater, the biosensor achieves a response time of 20 s during continual operation. The biosensor also maintains excellent detection stability and accuracy, with an RSD of ≤2.5% across various Cr(VI) concentrations under conditions of continual disturbance. These results reveal the biosensor’s potential for continual online monitoring of Cr(VI) concentrations in wastewater. Nonetheless, there are potential challenges in the long-term operational performance of this system, and future research will be necessary to further validate its stability.

## Figures and Tables

**Table 1:** The primer sequences utilized in the present study.

Primer Name	Primer Sequences (5′→3′)
Chr-Forward primer	
*Nco* I-ChrA-f	C↓C*ATGG*ATGAGCAAAACGGTCGTTCT
*Nco* I-ChrB-f	C↓C*ATGG*ATGCGTGTCTGGCGAACCCTGA
Chr-Reverse primer	
*Xho* I-ChrA-r	C↓*TCGAG*TTCTGCGCCGGACAGT
*Not* I-ChrB-r	GC↓*GGCCGC*TCACTCTGCGGAAGAACGA

….indicates restriction enzymes recognition sequences; ↓ indicates restriction enzymes cutting sites.

**Table 2:** Cr(VI) measurement in synthetic wastewater using the SCMFC-based biosensor in continual operation and atomic absorption spectroscopy, colorimetric method, and ion chromatography in batch operation.

	Inlet Cr(VI) Concentration (mg/L)				
	0.02	0.5	5	50	150
AAS ^1^	0.0213 ± 0.0024	0.502 ± 0.020	5.01 ± 0.03	52.6 ± 0.5	142.8 ± 1.26
Colorimetric method	0.0202 ± 0.0081	0.4981 ± 0.012	4.96 ± 0.15	47.3 ± 2.4	155.2 ± 3.71
Ion chromatography	0.0201 ± 0.0023	0.4982 ± 0.008	5.01 ± 0.08	51.9 ± 0.9	152.4 ± 0.82
SCMFC-based biosensor	0.0205 ± 0.0035	0.4967 ± 0.018	4.98 ± 0.07	51.2 ± 1.2	147.4 ± 0.96
Deviation (%) ^2^	6.5	0.4	0.2	5.2	−4.8
Deviation (%) ^3^	1	−0.38	−0.8	−5.4	3.47
Deviation (%) ^4^	0.5	−0.36	0.2	3.8	1.6
Deviation (%) ^5^	2.5	−0.66	−0.4	2.4	−1.73

^1^ Atomic absorption spectroscopy. ^2^ The value determined by atomic absorption spectroscopy in comparison to the standard concentration of Cr(VI). ^3^ The value determined by the colorimetric method in comparison to the standard concentration of Cr(VI). ^4^ The value determined by ion chromatography in comparison to the standard concentration of Cr(VI). ^5^ The value determined by the SCMFC-based biosensor in comparison to the standard concentration of Cr(VI).

## Data Availability

Data are contained within the article.
